# Glucose transporters: Important regulators of endometrial cancer therapy sensitivity

**DOI:** 10.3389/fonc.2022.933827

**Published:** 2022-08-05

**Authors:** Xing Zhang, Jia-Jing Lu, Ayitila Abudukeyoumu, Ding-Yu Hou, Jing Dong, Jiang-Nan Wu, Li-Bing Liu, Ming-Qing Li, Feng Xie

**Affiliations:** ^1^ Medical Center of Diagnosis and Treatment for Cervical and Intrauterine Diseases, Obstetrics and Gynecology Hospital of Fudan University, Shanghai, China; ^2^ Laboratory for Reproductive Immunology, Hospital of Obstetrics and Gynecology, Shanghai Medical School, Fudan University, Shanghai, China; ^3^ Department of Gynecology, Hospital of Obstetrics and Gynecology, Shanghai Medical School, Fudan University, Shanghai, China; ^4^ Clinical Epidemiology, Clinical Research Center, Obstetrics and Gynecology Hospital of Fudan University, Shanghai, China; ^5^ Department of Gynecology, Changzhou No. 2 People’s Hospital, affiliated with Nanjing Medical University, Changzhou, China; ^6^ National Health Commission (NHC) Key Lab of Reproduction Regulation, Shanghai Institute for Biomedical and Pharmaceutical Technologies, Fudan University, Shanghai, China; ^7^ Shanghai Key Laboratory of Female Reproductive Endocrine Related Diseases, Hospital of Obstetrics and Gynecology, Fudan University, Shanghai, China

**Keywords:** glucose transporter, endometrial cancer, proliferation, angiogenesis, apoptosis, therapy sensitivity

## Abstract

Glucose is of great importance in cancer cellular metabolism. Working together with several glucose transporters (GLUTs), it provides enough energy for biological growth. The main glucose transporters in endometrial cancer (EC) are Class 1 (GLUTs 1–4) and Class 3 (GLUTs 6 and 8), and the overexpression of these GLUTs has been observed. Apart from providing abundant glucose uptake, these highly expressed GLUTs also participate in the activation of many crucial signaling pathways concerning the proliferation, angiogenesis, and metastasis of EC. In addition, overexpressed GLUTs may also cause endometrial cancer cells (ECCs) to be insensitive to hormone therapy or even resistant to radiotherapy and chemoradiotherapy. Therefore, GLUT inhibitors may hopefully become a sensitizer for EC precision-targeted therapies. This review aims to summarize the expression regulation, function, and therapy sensitivity of GLUTs in ECCs, aiming to provide a new clue for better diagnosis and treatment of EC.

## Introduction

Endometrial cancer (EC) ranks as the sixth most common malignancy diagnosed among women. Most cases are diagnosed after menopause (1). However, morbidity has been increasing over the past years. Many risk factors are considered to be closely related to EC, such as obesity, estrogen exposure, insulin resistance, and age (2–4). Generally speaking, EC can be divided into two types. Type 1 refers to endometrioid carcinoma and accounts for 75%, which is believed to be closely associated with long-term estrogen stimulation, while type 2 tends to be high grade and with poor prognosis (5, 6). Moreover, approximately 5%–10% of endometrial carcinomas have a hereditary basis from hereditary non-polyposis colorectal cancer (7).

Glucose plays a deterministic role in cellular metabolism. Working together with several glucose transporters (GLUTs), it provides enough energy for biological growth through diffusion or secondary active transport. The major facilitator superfamily (MFS) of membrane transporters is encoded by *SLC2* genes, controlling the transmembrane movement of various substrates. As vital facilitative sugar transporters, these GLUTs can be categorized into three classes in the light of respective sequence similarity and substrate specificity: Class 1 (GLUTs 1–4 and 14), Class 2 (GLUTs 5, 7, 9, and 11), and Class 3 (GLUTs 6, 8, 10, 12, and 13/HMIT). Their physiological substrate is generally a hexose, but their substrates can be urate (8), dehydro-ascorbate (9), polyols (10), myo-inositol (11), and trehalose (12). Among this SLC2 family of 14 members, GLUT2/4/12 mainly functions as insulin-dependent transporters, GLUT5/7/11 chiefly refers to fructose transport (13–17), and GLUT6 is a lysosomal transporter (18). All in all, GLUTs 1–4 play a predominant role in maintaining cellular glucose uptake and functional metabolic homeostasis.

Various physiological functions of the proteins mainly depend on factors including the difference of principal substrates and the cell type distribution, and the relevance between the proteins and subcellular compartments. Some GLUT proteins can translocate between subcellular compartments, and this effectively promotes their control of long- and short-time scales in regulating the supply of glucose to tissues (19). The occurrence of their dysfunction means a number of pathological disorders, such as GLUT1 deficiency syndrome and the Fanconi–Bickel syndrome, type 2 diabetes mellitus, and cancers.

It is widely acknowledged that various malignant tumors have an activated glucose metabolic rate under some adverse circumstances, such as hypoxia, inflammation, and malnutrition (20, 21). However, a special pattern called the Warburg effect shows that even under an oxygen-rich environment, the glucose metabolism of tumor cells still remains quite active (22). Generally, a high expression of GLUTs accelerates glucose metabolism, which is indispensable for endometrial proliferation and differentiation (23). This review aims to summarize the expression regulation, function, and therapy sensitivity of GLUTs in EC.

## Glucose transporters in endometrial cancer

It has been reported that the main GLUTs in EC are mainly Class 1 (GLUTs 1–4) and Class 3 (GLUTs 6 and 8), and their basic functions have been mentioned above. Next, we will try to elaborate on the expression of these GLUTs in endometrial cancer (**Table 1**).

**Table 1:** Expression of glucose transporters in endometrial cancer.

Subtypes	Highly expressed localization			The relationship with grade/prognosis	
	Tissues	Stromal cells	Epithelial cell	Grade	Prognosis
GLUT1	+	+	+	+	Not mentioned
GLUT2	**+**	**+**	**+**	Not mentioned	Not mentioned
GLUT3	**+**	**+**	**+**	**+**	−
GLUT4	**+**	**+**	**+**	**+**	Not mentioned
GLUT6	**+**	**+**	**+**	**+**	**+**
GLUT8	**+**	**+**	**+**	**+**	**+**

+, expression or positive relationship; −, no relationship.

*Class 1 (GLUTs 1–4)*: GLUT1 protein mainly localizes in the luminal epithelium, glandular epithelium, endometrium stroma, and endothelial cells (24–29). Of note, either in healthy endometrium or EC tissues, the relative expression of GLUT1 is greater than that of any other GLUTs, suggesting that it is the most important transporter in endometrial tissues. In addition, studies have confirmed that the expression of GLUT1 is related to tumor differentiation. Compared with well-differentiated tumors, the expression of GLUT1 is significantly elevated in poorly differentiated tumors, which may be of great significance for predicting prognosis and survival estimates of EC (30). Previous reports have indicated that GLUT2 has a low expression in EC and is not controlled by a hormone, but it is not observed in normal endometrium (31, 32). The mRNA level of GLUT3 seems to be much lower than that of GLUT1 in EC. Compared with the expression of estrogen/progesterone receptor (ER/PR)-negative EC, that of GLUT3 in ER/PR-positive EC is much higher (23). However, the relationship between the expression of GLUT3 and prognosis in EC has not been clarified. A report has shown that GLUT4 is barely present in healthy endometrium; nevertheless, it is upregulated in EC and might have a similar level of expression to GLUT3 (24, 33).

*Class 3 (GLUTs 6 and 8)*: Moststudies show that the level of GLUT6 is quite low in normal endometrial epithelial and stromal cells, while it is upregulated in early-stage EC cells. Furthermore, the mutations and amplifications of GLUT6 are observed more frequently in EC than in any other malignancies. However, a study carried out by Byrne et al. showed that GLUT6 (instead of GLUT1) is the most significantly elevated GLUT in the malignant endometrium and is especially highly expressed in cancerous glandular epithelial cells, which are closest to blood vessels in the surrounding stroma. This finding indicates that GLUT6 is pivotal for the occurrence of EC and that it may have some other unknown functions that remain to be discovered (34). GLUT8 is predominantly localized in the endoplasmic reticulum and has a moderate expression level, which can translocate to the cell surface under insulin stimulation, assisting in the indispensable glucose consumption for glycosylation of protein. GLUT8 is highly expressed in EC. Similar to GLUT1, the expression of GLUT8 is also related to tumor differentiation, and a higher level is observed in poorly differentiated tumors. Of note, its expression reached a peak in endometrial serous carcinoma (35, 36). Interestingly, whether in mammary epithelial cells or 3T3-L1 adipocyte cells, the expression of GLUT8 seems to be reduced by hypoxia but is not affected by the small interfering RNA (siRNA) knockdown of hypoxia-inducible factor-1α (HIF-1α), indicating that hypoxia may not play a predominant role in regulating GLUT8 expression, which totally differs from other hypoxia-dependent increased GLUTs (GLUT1/3) (24, 37). However, whether its expression level in hypoxia-related endometrial cancer cells (ECCs) is similar to the level of these cells is unclear until now.

## Upstream regulators of glucose transporters

### Estrogen or progesterone

Most studies support that estrogen can significantly increase the expression level of GLUT1, far more than the effect on other GLUTs (**Table 2**) (32). Differing from traditional two nuclear ERs that function as ligand-activated transcription factors, G-protein-coupled ER 1 (GPER), formerly known as GPR30, has an increased expression in the intracellular location of various cancer cells (e.g., breast, ovaries, and ECCs) and becomes involved in transcriptional activities, such as the production of cyclic adenosine monophosphate (cAMP), phosphatidylinositol 3-kinase (PI3K)/serine-threonine kinase (AKT), and AMP-activated protein kinase (MAPK) pathways, which indirectly strengthen the combination between ERs and other transcriptional factors (38). High levels of GPER expression are in consistence with poor survival (39). Here these estrogen-induced GLUTs may be achieved by activating independent transcription GPER or its downstream factor 6-phosphofructo-2-kinase/fructose-2,6-biphosphatase 3 (PFKFB3) levels in human healthy endothelial cells (40, 41). Progesterone also has a role in promoting the expression of GLUT1, while this effect is not as strong as that of estrogen. However, the combination treatment of estrogen and progesterone eliminates the two effects; namely, the combination therapy of the two hormones reduces the expression of GLUTs. Upregulated GLUT4 is predominantly associated with estrogen combined with its receptor, which poses an important role in the epithelial–mesenchymal transition (EMT) process of EC by stimulating vascular endothelial growth factor (VEGF) secretion (42). The expression of GLUT4 can also be elevated *via* estrogen-induced ESR1 regulation, which may be through SRC (proto-oncogene tyrosine-protein kinases)-mediated phosphorylation of ESR1 in normal muscle and adipose cells (43). In type 2 diabetes, this may induce GLUT4 expression and plasma membrane GLUT4 translocation in adipocytes (44). Another study shows that estrogen indirectly activates primary gene transcription-specificity protein 1, which directly functions as the GLUT4 gene promoter, increasing the expression of GLUT4 in type 2 diabetes (45). However, recent studies show that both estrogen and progesterone have little influence on the activity of the low-affinity transporter GLUT2/3/6.

**Table 2:** Upstream regulators of GLUTs in endometrial cancer.

Upstream regulators		GLUT1	GLUT3	GLUT4	GLUT6	Regulatory mechanisms
Hormones	Estrogen	+		+		ER
	Progesterone	+				PGRMC1, IR
High insulin		+		+		IRAP, IGF1R, PI3K/AKT pathway
High glucose		+	+	+		ER, AMPK/mTOR/S6 pathway
Hypoxia		+	+			HIF-1α, ATP, HtrA3
Cytokines	IL-3/IL-7	+				PI3K/AKT/mTOR pathway
	TNF-α				+	NF-κB, RELA
	VEGF	+				/
Enzymes	ABHD5	+				AKT pathway
	ALDH	+				/
Natural compounds	Flavonoids	−				/
	Vitamin C	−				HIF-1α

+, positive regulation; −, negative regulation.

HIF-1α, hypoxia-inducible factor-1α; VEGF, vascular endothelial growth factor; IL-3/IL-7, interleukin 3/7; ABHD5, abhydrolase domain containing 5; ALDH, high-level aldehyde dehydrogenase; Vitamin C, ascorbic acid; ER, estrogen receptor; PGRMC1, the progesterone receptor membrane component 1; IR, insulin receptor; IRAP, insulin-regulated aminopeptidase; IGF1R, insulin-like growth factor 1 receptor; PI3K, phosphatidylinositol 3-kinase; AKT, the serine-threonine kinase; AMPK, adenosine 5′-monophosphate (AMP)-activated protein kinase; mTOR, mammalian target of rapamycin; ATP, adenosine triphosphate; HtrA3, high-temperature requirement A3; NF-κB, nuclear factor kappa-B.

As is commonly known, long-time estrogen exposure is closely associated with hyperplastic proliferation of the endometrial glands; however, the effect of progesterone is quite the opposite. There is multiple evidence elucidating that progesterone plays an antagonistic role in inhibiting cell growth, invasiveness, and differentiation in type 1 EC (46). The progesterone receptor membrane component 1 (PGRMC1) is the first identified progesterone-binding membrane protein and has a high expression in various cancer cells. PGRMC1 can stimulate the expression of insulin receptors (IRs) in the plasma membrane and increase the level of GLUT1 and GLUT4 in the plasma membrane (47). However, whether the upregulation of GLUT1/4 is induced by IR remains uncertain.

### High insulin and high glucose

Insulin can increase the expression, transport, and oxidation of GLUT1. In pancreatic cancer cells, insulin can stimulate DNA synthesis by activating PI3K and phosphatidylinositol kinase (PIPK) in a concentration- and time-dependent manner (48). Previous studies have suggested that insulin can activate the PI3K/AKT pathway in muscle and adipose cells, which shows a beneficial effect on GLUT4 vesicle trafficking to the cell membrane (49). Furthermore, placental leucine aminopeptidase (P-LAP) is a cell surface aminopeptidase and a synonym for oxytocinase, referred to as insulin-regulated membrane aminopeptidase (IRAP). Hyperglycemia or hyperinsulinemia can be a signal to facilitate GLUT4 expression and PI3K/AKT pathway, which can be mediated by P-LAP/IRAP pathway (50, 51). A study has shown that insulin-like growth factor 1 receptor (IGF-1R) can inhibit the translocation of GLUT8 to the cytoplasm, achieving redistribution of cell survival in the murine blastocyst. Nevertheless, how insulin regulates GLUT8 expression in EC has not been reported so far (52).

High glucose directly activates the expression of GLUT1 and GLUT3 *via* the modulation of AMPK/mammalian target of rapamycin (mTOR)/S6 signaling in ECCs (53). Another study found that high glucose can upregulate the level of ER-mediated GLUT4 and facilitate the expression of VEGF/VEGFR, which in turn increases the viability and invasion of ECCs (42).

### Hypoxia

Hypoxia increases the expression of GLUT1 and GLUT3 in endometrial stromal cells (54, 55). It seems that hypoxia can increase intracellular adenosine triphosphate (ATP), which sequentially triggers GLUT1 translocation to the plasma membrane *via* ATP-sensitive potassium channels (KATP channels) (56). Some studies have confirmed that glucose consumption increases significantly under hypoxic conditions, and the key regulator HIF-1α seems to play an important role in the process. In both type 1 and type 2 EC, HIF-1α is widely expressed in epithelial and stromal components; however, its role may vary. By activating its downstream genes such as GLUT1, VEGF, and epidermal growth factor (EGF), HIF-1α accelerates the occurrence and development of EC (57, 58). Nevertheless, it has been reported that HIF-1α activity can be constitutively induced by oxygen-insensitive pathways in addition to its induction by hypoxia/anoxia, such as ubiquitination, acetylation, sumoylation, hydroxylation, and phosphorylation. These pathways may make a joint effort to promote HIF-1α activity and expression, which further activate GLUT1-induced glucose uptake and crucial genes including VEGF and matrix metalloproteinases (MMPs). This mechanism has been reported in various cancers except in EC (59, 60). Increased SHARP1 is a physiological transcription factor to decrease the levels of HIF-1α, VEGF, and so forth, functioning as a protective molecule to repress the development of EC (61). This indicates that it may indirectly inhibit the expression the GLUT1 *via* in a HIF-1α-dependent manner. High-temperature requirement A3 (HtrA3) is a member of the ATP-independent serine protease family. Many studies have demonstrated that the expression of HtrA3 downregulates in some cancers, indicating that it may act as a pro-apoptotic protein in carcinogenesis (62). Hypoxia can further reduce the expression of HtrA3, promoting the development of EC (63). It has been reported that in vulvar squamous cell carcinoma, the expression of GLUT1 may be dependent on neither hypoxia nor aerobic glycolysis. However, this mode seems to be crucial for protecting DNA’s integrity from oxygen radical damage as well as promoting the regeneration of membranes (64).

### Various cytokines

Cytokines play an important role in synthesizing GLUTs. Furthermore, previous evidence supports that all the known risk factors for EC are directly or indirectly involved in inflammation pathways, such as estrogen, obesity-related factors, and diabetes mellitus (65). Tumor necrosis factor-α (TNF-α), one of the most powerful cytokines, may activate the downstream mediator-RELA *via* the nuclear factor kappa-B (NF-κB) signaling pathway, increasing the expression of GLUT6, in addition to enhancing local estrogen synthesis, insulin resistance, and the like (66). There is evidence that TNF-α may act alone or together with GLUT6, promoting the occurrence and progression of obesity-related EC (67). A high expression of TNF-α seems to play a part in some advanced and worse overall survival EC. Interleukin-3 (IL-3) activates the PI3K/AKT/mTOR pathway, promoting the activity, recycling, and internalization of GLUT1 in lymphoid/myeloid hematopoietic precursor cells (68). Interleukin-7 (IL-7) can upregulate reactive oxygen species, which rely on PI3K/AKT/mTOR pathway, upregulating GLUT1 expression in EC (69). In one previous EPIC cohort study, Dossus et al. concluded that IL-6 was associated with an increased risk of EC in obese women in addition to TNF-α (70). Trabert et al. confirmed the positive correlation between these cytokines (e.g., adipokines, inflammatory cytokines, and angiogenic factors) and obese women with EC in a nested case–control study (71). Further, with the VEGF-A becoming the most important component of the VEGF family, both VEGF-A binding to VEGFR1/2 and GLUT1 can be activated by HIF-1α under hypoxic conditions, accelerating the occurrence and development of EC (60, 72). Similarly, there exists a broad consensus that VEGF-A stimulated by estrogen also has a positive correlation with GLUT4 in promoting the angiogenesis process of EC. Sahoo et al. investigated visceral adipocytes that could induce VEGF activation in the angiogenesis of EC (73).

### Other regulators

Abhydrolase domain containing 5 (ABHD 5) acts as a carcinogenic component and is overexpressed in EC, which significantly increases the expression of GLUT1 and glycolysis enzymes *via* the AKT pathway, which is believed to be closely related to cell proliferation, invasion, and EMT in EC (74). High-level aldehyde dehydrogenase (ALDH) expression is significantly correlated with increased expression of GLUT1, and the glycolytic pathways’ activation plays a crucial role in the prognostic evaluation of EC (75). As natural compounds with antiproliferative activities, flavonoids can regulate the expression of GLUT1 and glucose uptake, which may be of help in controlling the growth of prostate cancer cells (76). Ascorbic acid (also named vitamin C) can downregulate the activity of many GLUTs, including GLUT1/3/4, which may be due to its repressive effect on HIF-1α under normoxic or hypoxic conditions. This mechanism has been delineated in many tumors, such as pancreatic cancer, liver cancer, and ovarian cancer (77).

## The functions of glucose transporters in endometrial cancer

### Cell proliferation and apoptosis

Increased GLUT1 is always associated with cell proliferation and tumorigenesis in various tumors *via* glucose supply (78–80). Additionally, GLUT1 was regarded as a downstream target of miR-150-5p, which can protect cells by inhibiting GLUT1 expression (81). Another report indicates that overexpression of GLUT1 is regulated by lncRNA-plasmacytoma variant translocation 1 (PVT1) through the PVT1/miR−150−5p/GLUT1 signaling axis to promote cell proliferation and invasion and inhibit apoptosis in oral squamous cell carcinoma (82). It has been reported that the expression of GLUT1 is positively related to that of CASC9 (a long non-coding RNA). In laryngeal carcinoma cells, through activating PI3K/AKT/mTOR and EGFR signal pathways, CASC9 facilitates cell proliferation and inhibits cell apoptosis (83), while the level of GLUT1 is also regarded as an independent prognostic predictor.

**Figure 1:**
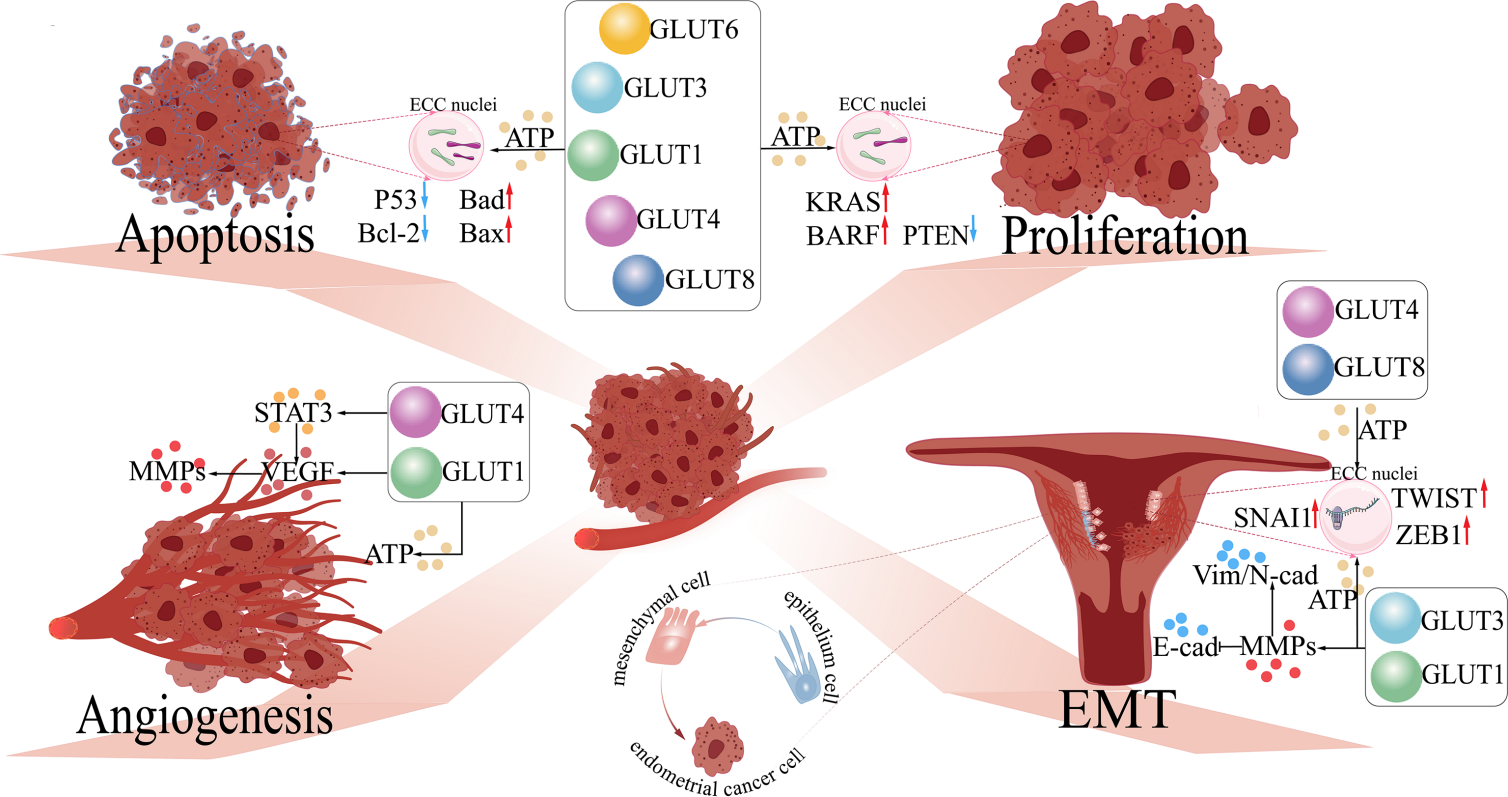
The functions of GLUTs in endometrial cancer. 1) Proliferation: GLUTs upregulate the expression of oncogenes (e.g., KRAS and BARF) and inhibit anti-oncogenes (e.g., PTEN) in endometrial cancer cells (ECCs) by providing abundant ATP for cellular metabolism. 2) Apoptosis: GLUTs downregulate pro-apoptosis genes (e.g., P53 and Bcl-2) and upregulate anti-apoptosis genes (e.g., Bad and Bax) in ECCs. 3) Angiogenesis: GLUT1 regulates VEGF and its downstream molecule (MMPs), further accelerating angiogenesis in ECCs by activating STAT3. 4) EMT: GLUT1/3 regulates the expression of EMT-related proteins (Vim, N-cad, and E-cad) by upregulating the levels of MMPs in ECCs, facilitating the development of EMT in ECCs; GLUT4/8 activates EMT-related transcription factors (TWIST, SNAI1, ZEB1) of ECCs. GLUTs, glucose transporters; STAT3, signal transducer and activator of transcription 3; VEGF, vascular and epidermal growth factor; MMPs, matrix metalloproteinases; EMT, epithelial–mesenchymal transition; Bcl-2, B-cell lymphoma-2; Bad, Bcl-2 agonist of cell death; Bax, BCL2-associated X; KRAS, Kirsten rat sarcoma; BARF: *Bam*HI A right frame 1; PTEN, phosphatase and tensin homolog; TWIST, time without significant symptoms of toxicity protein; SNAI, Snail-1 protein. .

Previous studies show that ECCs rely on complicated macromolecule synthesis to promote cell proliferation. Through stimulating different GLUT (GLUT1/3/6) production, the inactivation of proto-oncogenes phosphatase and tensin homolog (PTEN) and mutation of oncogenes (e.g., BRAF and KRAS) occur in the endometrium (**Figure 1**), activating the PI3K/AKT pathway (84, 85). GLUT1-related glucose uptake is tightly associated with ATM (an insulin-responsive protein kinase). As a crucial regulator of tumorigenesis, ATM facilitates the production of ATP and the activation signaling of AKT, promoting cell proliferation and inhibiting apoptosis in aggressive breast and prostate cancer cells (86).

Another study has also elucidated that GLUT3 expression can be upregulated by the Hippo-Yes-associated protein (YAP) at a transcriptional level. AMPK directly phosphorylates YAP at S61 and inhibits YAP transcriptional activity, maintaining glucose homeostasis in HEK293T and HeLa cells (87). In liver cancer, therapy with the sodium-glucose transporter 2 (SGLT2) inhibitor induces AMPK/mTOR-mediated cell cycle arrest, antiproliferation, and apoptosis, which may be regarded as a novel way of treatment. Nevertheless, the expression of SGLY2 has not been identified in EC (88). Estrogen is greatly associated with the expression of GLUT1/4. It is reported that estrogen has positive effects on excessive cellular proliferation and tumor differentiation *via* the dysregulation of Wnt signaling-related molecules including secreted frizzled-related protein 1 (sFRP1) and sFRP4 and the upregulation of the IGF pathway (32, 89). Furthermore, studies show that GLUT4 is significantly correlated with IRAP, which induces the activation of the PI3K/AKT pathway (4, 51, 90). Increasing evidence supports that activated GLUT1/4 expression and translocation from the cytoplasm to the membrane are positively induced by AKT, which may facilitate cell proliferation and lead to drug resistance in EC treatment (91). GLUT6 plays the most important role in glucose transport and glycolytic–lipogenic metabolism, providing glucose for ECCs (34, 92, 93). GLUT8 plays a key role in glucose supply by supporting serine/glycine biosynthesis of KRAS/the Kelch-like ECH-associated protein 1 double mutants in non-small cell lung cancer, while it has also been found to be importantly upregulated in EC (94).

### Epithelial–mesenchymal transition

Studies have confirmed that EMT is closely related to the occurrence, progression, metastasis, and even treatment resistance in EC. An early report indicated that estrogen-induced GLUT1/4 plays a crucial role in the malignant transformation of benign epithelial EC (95). Further, the expression of ER-related GLUT4 can be upregulated by high glucose, which in turn activates the transcription of many EMT-related molecules in EC (42). By providing abundant glucose, they meet the glucose metabolism demand of these tumor cells, which are far from stromal blood vessels (86). Estrogen activates the binding of ERα to the expression of ubiquitin-conjugating enzyme E2C promoter region and negatively modulates the expression of p53 (96). In addition, this combination can also improve the level of miR-200c, which inhibits the expression of PTEN and PTENP1, leading to the activation of the PI3K/AKT pathway (97). In a later study, the activation of the PI3K/AKT/mTOR pathway and the inhibition of the level of E-cad under the estrogen stimuli are both involved in the EMT process of EC (98). These mechanisms facilitate cell migration, invasion, and EMT-related vimentin in EC. Collectively, GLUTs may play a pivotal role in the process of EMT in an estrogen-dependent manner in EC.

There is increasing evidence that hypoxia is one of the microenvironmental factors that directly promote the EMT process and GLUT production in multiple cancers (99, 100). The expressions of glycolysis-related GLUT1/3 and EMT-related proteins (Vim, N-cad except for E-cad) are both increased under hypoxia conditions (101–103). A study concerning laryngeal carcinoma showed that by regulating the activity of MMPs, hypoxia-induced GLUT1/3 may induce EMT and promote cell invasion and metastasis (104). In addition, some studies showed that hypoxia-induced GLUT1 expression not only is closely correlated with tumor proliferation and angiogenesis but also has a strong positive correlation with Ki-67 expression (EMT-related) in epithelial ovarian carcinoma and diffuse large B-cell lymphoma (105, 106). Although such a mechanism has not been found in EC, we speculate that hypoxia-induced GLUTs may also participate in the regulation of EMT-related factors (such as MMP, SNAI1, TWIST, and ZEB1).

High glucose and estrogen can upregulate GLUT4, facilitating the expression of VEGF/VEGFR and the progression of EMT, which finally improves the viability and invasion of ECCs (42). As mentioned, the overexpression of GLUT8 is related to the differentiation, proliferation, and invasion of EC. Evidence suggests that the abnormal transposition of GLUT8 is significantly associated with the malignant transformation of ECCs. There are three possible mechanisms that might explain this phenomenon: intracellular phosphorylation events similar to those of GLUT4, mutation of the di-leucine motifs, and the inhibition of IGF-1R by antisense oligonucleotides (35).

### Angiogenesis

Angiogenesis acts as a critical part of tumor growth and invasion, providing a new colony for tumor immune escape (72). Factors participating in angiogenesis include fibroblast growth factor, VEGF, platelet-derived growth factor, and EGF. Among them, VEGF plays the most important role (107). As mentioned before, in EC, the expression of GLUT1 is related to tumor differentiation; the expression of GLUT1 is significantly elevated in poorly differentiated types compared with well-differentiated types. In several EC-related clinical studies, tumor aggressiveness has a positive correlation expression of GLUT1 in patients with early EC; nevertheless, the aggression-related molecules of GLUT1 have not been identified (108). Further studies confirm that the expression of GLUT1 is positively correlated with VEGF and its downstream component MMPs, which is induced by HIF-1α (60).

A large amount of evidence shows a tight relationship between estrogen and angiogenesis and that estrogen can activate the PI3K/AKT signaling pathway in a HIF-1α-dependent manner, sequentially stimulating VEGF and GLUT1/4 expression levels (109, 110). As mentioned above, we speculate that SHARP1(a basic helix-loop-helix transcription repressor) may indirectly regulate GLUT1 overexpression and VEGF levels *via* a HIF-1α-dependent manner, which is negatively associated with hypoxia-related angiogenesis in EC (61). In addition, GLUT4 induced by high glucose or estrogen may also play a role in promoting cell proliferation and endothelial cell migration. Through activating the signal transducer and activator of the transcription 3 (STAT3) signaling pathway, GLUT4 upregulates the expression of VEGF (42, 111, 112). We speculate that GLUT1/4 may promote the invasion ability and angiogenesis in EC by influencing VEGF and its downstream gene, although the exact mechanism remains unknown (113–115).

As mentioned before, progesterone can increase the expression of GLUT1. Studies show that through activating the GLUT1-related PI3K/AKT pathway, progesterone can also downregulate the expression of progesterone receptor B (PRB), promoting the proliferation and angiogenesis of ECCs (116).

## Glucose transporters in endometrial cancer therapies

### Glucose transporter-related radiotherapy sensitivity

**Figure 2:**
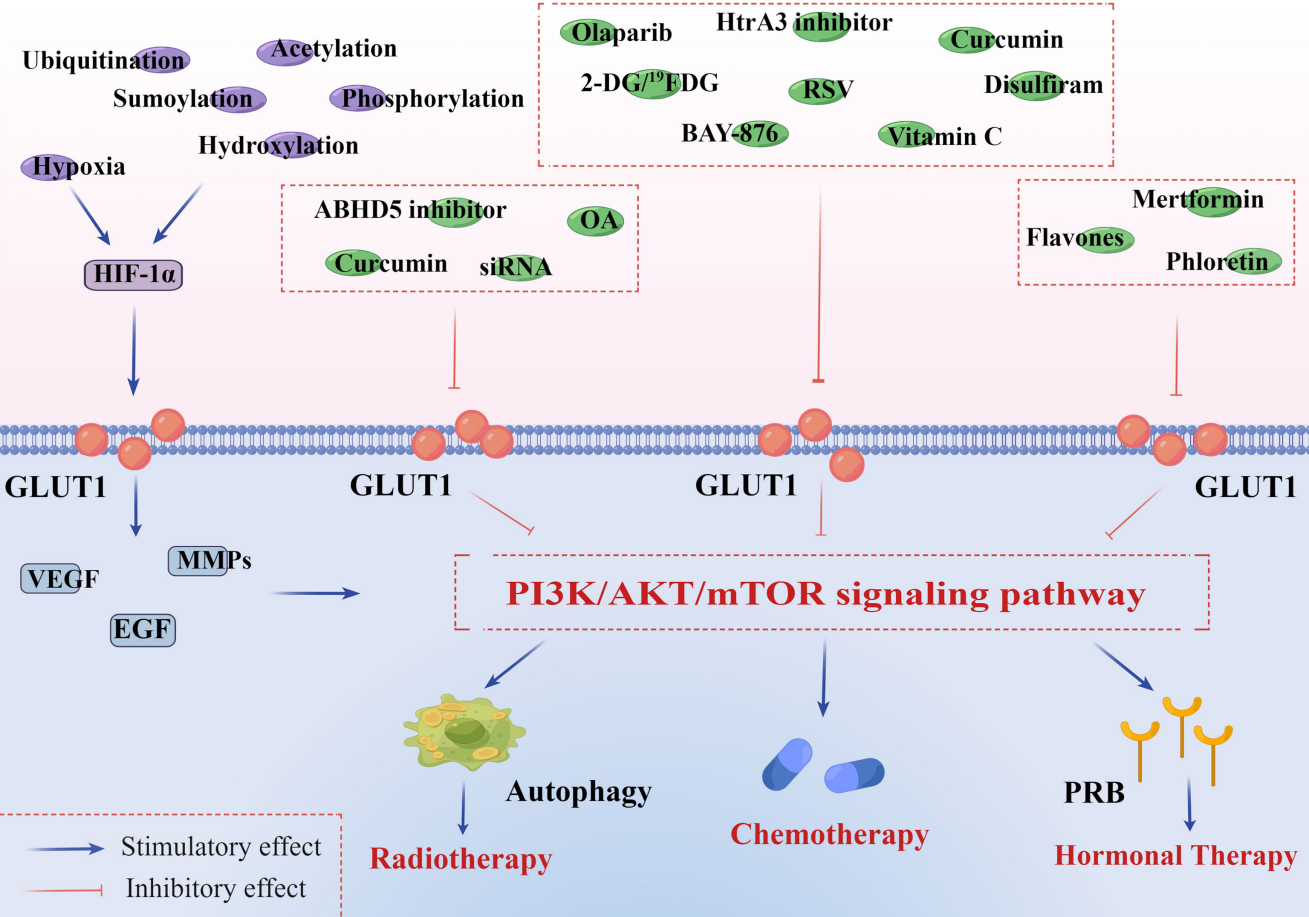
PI3K/AKT/mTOR signaling pathway is associated with GLUT1 overexpression and endometrial cancer therapies. HIF-1α is implicated in both GLUT1 expression and aberrant PI3K/AKT/mTOR signaling pathway in tumor microenvironments. In addition to hypoxia, HIF-1α can be constitutively induced by oxygen-insensitive pathways, such as ubiquitination, acetylation, sumoylation, hydroxylation, and phosphorylation. These pathways jointly promote the expression of HIF-1α and further activate the downstream genes of GLUT1, including VEGF, EGF, and MMPs. 1) Radiotherapy: curcumin inhibits a site overlapping the cytochalasin B of GLUT1 and metabolism-related enzymes. OA decreases the expression of many significant factors, including GLUT1, HIF-1α, Ki67, and P53. GLUT1-siRNA interferes with the targeted gene, inhibiting the synthesis of GLUT1. ABHD 5 plays an oncogenic role in the development of EC, and its knockdown can notably suppress ECC proliferation and invasion *in vivo*. All of them can inhibit the PI3K/AKT/mTOR pathway and activate autophagy of endometrial cancer cells (ECCs), increasing the sensitivity to radiotherapy. 2) Chemotherapy: olaparib inhibits the activity of GLUT1 in plasma in a concentration-dependent manner. BAY-876 can suppress cell viability and decrease stemness oncogene (Nanog and c-Myc) expression of ECCs. RSV inhibits GLUT1-induced glycolysis in a PI3K/AKT/mTOR-dependent manner, enhancing the anti-endometrial cancer (anti-EC) effects of cisplatin and doxorubicin. Curcumin inhibits a site overlapping the cytochalasin B of GLUT1 and metabolism-related enzymes. Vitamin C inhibits the expression of HIF-1α and GLUT1 in ECCs. 2-DG/^19^FDG and disulfiram show notably antiproliferative and anti-angiogenesis effects by downregulating the level of GLUT1. Targeting HtrA3 can enhance the cytotoxic effect of chemotherapy *via* the X-linked inhibitor of apoptosis protein cleavage. 3) Hormonal therapy: GLUT1 participates in regulating PR of ECCs in a PI3K/AKT/mTOR pathway-dependent manner. Flavones, phloretin, and metformin can greatly increase the sensitivity of hormonal therapy in EC by strengthening PR transcriptional activity. This figure was drawn by Figdraw (www.figdraw.com). GLUT1, glucose transporter 1; HIF-1α, hypoxia-inducible factor-1α; VEGF, vascular and epidermal growth factor; EGF, epidermal growth factor; MMPs, matrix metalloproteinases; OA, oleanolic acid; siRNA, small interfering RNA; ABHD 5, abhydrolase domain containing 5; RSV, resveratrol; DG, deoxyglucose; PR, progesterone receptor.

Radiation therapy is one of the important complementary treatments for EC. In recent years, research on how to enhance the sensitivity of radiation therapy in EC has received more and more attention. Autophagy is of great importance in the formation of radiotherapy sensitivity in solid tumors, and studies have verified that the PI3K/AKT/mTOR signaling pathway may negatively regulate intracellular autophagy (117, 118). GLUT1 siRNA is the targeted inhibitor of GLUT1-mediated glucose uptake, which can increase radiosensitivity through activating autophagy in a PI3K/AKT pathway-dependent manner and reducing DNA repair capability (**Figure 2**) (119, 120). In addition, it can also interfere with the active glucose metabolism to a large extent. This mechanism has been certified in laryngeal carcinoma and prostate cancer (121, 122).

The overexpression of GLUT1 is closely related to the radiation therapy resistance in EC. It has been reported that in rat glioma tumor cells, oleanolic acid (OA) shows a radio-sensitizing effect by decreasing the expression of many significant factors, including GLUT1 and its upstream molecule HIF-1α, Ki-67, and P53 (123).

A large amount of data indicate that the anti-radiosensitivity in EC refers to PI3K/PTEN/AKT/mTOR signaling pathway, MAPK signaling pathway, and NF-kB signaling pathway; each of them is directly or indirectly involved in tumor radio-resistance and GLUT1-induced malignant processes that include proliferation, angiogenesis, and EMT in EC (124, 125). It is clear that either upstream elements’ inhibitors of these significant pathways or inhibitors of GLUT1 itself should play a therapeutic role in the radio-resistance of EC. Among them, a prominently activated PI3K/AKT/mTOR pathway could increase the expression and translocation to the plasma membrane of GLUT1/3, and numerous preclinical setup and clinical trials have been launched with some of their inhibitors approved to be used in trials (126). For example, using sunitinib (one AKT inhibitor) as a neoadjuvant treatment could promote autophagy along with radiosensitivity to recalcitrant EC, which only provides a novel point for clinical implementation of sunitinib (127).

In the tumor microenvironment, hypoxia, high glucose and insulin, and various cytokines and all their inhibitors could play different roles in the inhibition of the expression of GLUTs and the final development of EC. Both hypoxia and its inducible factor HIF-1α known as an aggressive biomarker are positively correlated with the expression of GLUT1 and VEGF, which makes their inhibitors a great potential treatment in the prevention of angiogenesis, EMT process, and increased radio-sensitivity of EC (128). As a HIF-1α inhibitor, curcumin (diferuloylmethane) has been assumed to decrease glucose uptake in many cancers, such as lung, cervical, prostate, and breast cancers. It might function as a novel anticancer drug to assist chemoradiotherapy by inhibiting a site overlapping the cytochalasin B of GLUT1 and metabolism-related enzymes (129–131). In some studies concerning cervical cancer, the theory that curcumin enhances radiosensitivity by inhibiting GLUT1-induced PI3K/AKT/mTOR pathway or MAPK/mTOR/ULK1 pathway and subsequently activating autophagy has also been confirmed (121, 125, 132). Therefore, we speculate that there may be a similar mechanism in EC. Several studies also indicate that P-LAP siRNA (an inhibitor of hyperinsulinemia) may be a potential agent of molecular-targeted therapy for EC *via* the downregulation of GLUT4 expression and the prevention of tumor cells’ proliferation and angiogenesis (133). As mentioned before, ABHD5 may play an oncogenic role in GLUT1 expression and the EMT process of EC *via* the AKT pathway, and its knockdown notably suppresses tumor cell proliferation and invasion *in vivo*. This illustrates that it may act as a potential therapeutic target in radio-resistance of EC (74).

Of course, large amounts of cytokines also play a unique role in EC. Among them, VEGF as an upstream regulator in PI3K/AKT/mTOR signaling pathway illustrates that either its inhibitors or mTOR inhibitors could be a valid treatment of angiogenesis and EMT of EC and the level of GLUT1 (73). Likewise, in the epithelial cells, TNF-α also efficiently binds to its receptor to activate the NF-κB transcription factor and subsequently regulates the expression of genes and Snail-like proteins, which control E-cadherin transcription in tumor invasion (134). TNF-α inhibitors (e.g., adalimumab) are known as effective agents in both suppressing the level of GLUT6 and restraining the proliferation, angiogenesis, and EMT process in EC (66, 135). Therefore, it is possible that not only GLUT1 inhibitors but also blocking crucial pathways or upstream regulative factors (e.g., hypoxia, hyperinsulinemia, and cytokines) could promote apoptosis procedure and radiosensitivity in EC.

### Glucose transporter-related chemotherapy sensitivity

The resistance of tumor cells to drugs is a major obstacle in cancer chemotherapy. Thus, GLUTs as a novel therapeutic target might be of great importance in the chemotherapy of EC (136). At present, there are four inhibitor-bound hGLUTs, hGLUT 1–4, and each of them can provide a significant inhibitory effect on glucose uptake and cancer cell proliferation (137). BAY-876, as a specific inhibitor of GLUT1, can suppress ALDH-dependent glycolytic activation, cell viability, and stemness marker (e.g., Nanog and c-Myc) expression in ALDH-high ECCs. It can strongly suppress the proliferation of endometrial cancer stem cells (CSCs) when used in combination with paclitaxel (75). However, despite some current progress in the treatment of hepatocellular carcinoma, administration or intravenous infusion of BAY-876 can cause the drug to be distributed systemically, which greatly interferes with the physical uptake of glucose in the body in addition to the negligible dose distribution at the lesion site of cancer cells (138, 139).

As mentioned before, ALDH also plays an important role in the maintenance of CSCs and chemoresistance through upregulating GLUT1-induced glycolysis. Of note, the pan-ALDH-specific inhibitor disulfiram (DSF) can improve the paclitaxel-resistance effect in EC by suppressing GLUT1 and crucial pathways in several processes (e.g., proliferation, EMT, and angiogenesis) (75). Although it has not been confirmed in EC treatment, this combination therapy has already been applied in many preclinical trials for the treatment of several other cancers (e.g., non-small cell lung cancer, glioblastoma, and breast cancer) (140, 141). Pharmacodynamics reveals that DSF has a risk of reversible neurological toxicities, which readily occur after treatment with 1,000 mg per day (142).

Olaparib is an inhibitor of poly(ADP-ribose) polymerase (PARP)-1/2/3 (143–145). It can inhibit the activity of GLUT1 in plasma in a concentration-dependent manner and reduce the expression of cyclin D1 *via* a PARP-1 level-dependent manner. In several EGFR inhibitor-resistant cancers, such as glioblastoma and lung cancer, olaparib reduces lactate production and glucose uptake in a pyruvate kinase 2 (PKM2)-dependent manner (146). The most common adverse effects are nausea, fatigue, anemia, and vomiting (147).

Substantial work has constantly sought to target glucose metabolism. Among them, directly downregulating glucose levels through a special compound known as 2-deoxyglucose (2-DG) or inhibiting lactate production and excretion could be more prominent than others. 2-DG has been used for antiproliferation in numerous preclinical studies and partial clinical testing. The example here is that 2-DG shows notable antiproliferative effects and increased sensitization of resistant cells on oral cancer by downregulating the level of PARP, LDHA, and GLUT1 when it is used in a combination therapy with paclitaxel and erlotinib (148). Nevertheless, how to manage serious hypoglycemia symptoms caused by its higher dose along with an insufficient therapeutic response to its lower dose limits its clinical efficacy (149). Thus, ^19^FDG, as an alternative to 2-DG, shows a better ability to inhibit GLUT-dependent glycolysis, prevent cell viability and proliferation, and induce apoptosis *in vitro* under normoxic and hypoxic conditions. Niccoli et al. confirmed it in HeLa cells *via* by combining ^19^FDG with doxorubicin and comparing its efficacy with that of 2-DG and doxorubicin (150). Excessive lactate production could promote angiogenesis and tumor vascularization through the induction of HIF-1α-stimulated VEGF increase, and dysregulated pH is also involved in chemotherapeutic drug resistance (e.g., vinblastine, doxorubicin, and paclitaxel) (151). The cardiac Na^+^/H^+^ exchanger (NHE1) is a membrane glycoprotein for multiple housekeeping tasks relying on cell function, including regulation of intracellular pH, Na^+^ concentration, and cell volume. Therefore, clinical NHE1 inhibitors and much more selective inhibitors (e.g., KR-33028 and cariporide) might be taken into consideration to attain increased chemotherapeutic effectiveness in EC; this has been assessed in a triple-negative breast cancer model (152). It is proven to be well tolerated in people with cardiovascular disease. However, some side effects are inevitable, mainly related to drug accumulation and cerebrovascular complications (153).

Resveratrol (RSV) has no effect on GLUT1 mRNA and protein expressions but disturbs intracellular GLUT1 trafficking to the plasma membrane by suppressing AKT/mTOR activation, which ultimately impairs glucose uptake and induces apoptosis in ovarian cancer cells (154). In EC, RSV can enhance the antitumor effects of cisplatin and doxorubicin in a time-dependent manner (155–157). It may regulate the expression of EGF/VEGF and angiogenesis to promote chemosensitivity in an estrogen-dependent or estrogen-independent manner (158, 159). This inhibitor has been controversial because its biologically effective concentration is hard to confirm. Moreover, it acts as a natural reservoir for body antioxidants and is accompanied by many toxic effects, such as high dosage-associated hormetic effects, systemic inhibition of P450 cytochromes, and attenuation of the activities of drugs (160).

Curcumin analog (EF24) could exert antiproliferative and anti-angiogenic effects on three ovarian cancer cells (SKOV-3, A2780, and OVCAR-3) *in vivo via* the downregulation of GLUT1-related glucose glycolysis, lactate production, and its upstream molecule HIF-1α (161). Anti-GLUT1 antibody or curcumin combined with doxorubicin could significantly enhance the ability in killing colorectal adenocarcinoma cells (162). This result leads us to speculate that both curcumin and anti-GLUT1 antibody may also have the effect of sensitizing chemotherapeutic drugs of EC (121, 132). It is reported that curcumin can cause diarrhea, and other toxic and adverse effects have not been confirmed. However, in the long-term rat trials, adverse effects are noticeable, such as incidence of ulcers, chronic inflammation, and hyperplasia of the cecum as well as carcinogenesis (163).

Ritonavir displays inhibitory effects on GLUT4 expression and induces the apoptosis of multiple myeloma (MM) cells by reducing myeloid cell leukemia-1 expression. It is regarded as a sensitizer in the therapy of MM, which can make tumor cells more sensitive to drugs such as doxorubicin, dexamethasone, and melphalan (164, 165). Patients who are treated with ritonavir at a dose higher than 7.9 ml/L may be at a higher risk of experiencing neurological or gastrointestinal side effects. Although ritonavir’s sensitizing effect in the chemotherapy of EC has not been reported so far, related studies have already been carried out in many clinical trials for the treatment of cancers, such as multiple myeloma, prostate cancer, and breast cancer (166, 167).

Vitamin C plays an important role in VEGF-related angiogenesis and anti-chemoresistance in many cancers by inhibiting the expression of HIF-1α and GLUT1/3/4 (77, 168). For example, through inhibiting extracellular signal-regulated kinase 1/2 and PKM2 phosphorylation, the combination of vitamin C and cetuximab can significantly downregulate the expression of GLUT1 in KRAS colon cancer (169). Therefore, we infer that perhaps vitamin C is also valid in enhancing the sensitivity of chemotherapy in EC. Targeting HtrA3 might be a potential therapeutic measure to reverse the negative effects induced by hypoxia and enhance the cytotoxic of conventional chemotherapy *via* the X-linked inhibitor of apoptosis protein (XIAP) cleavage in EC (170). However, a more detailed understanding of the molecular mechanisms and cellular targets in clinical treatment agents is needed.

### Hormonal therapy sensitivity

Progestogen is the most commonly used drug in the conservative treatment of early EC (171, 172). However, the response rate to progestin therapy varies from person to person. As mentioned before, GLUT1 expression is positively correlated with the activation of PI3K/AKT pathways. Recent evidence has implicated that PI3K/AKT pathway increases the drug resistance of progestin in EC by weakening PRB transcriptional activity. GLUT1 may become involved in regulating PR in the PI3K/AKT pathway-dependent manner; therefore, GLUT1 inhibitors may be an effective therapeutic strategy for increasing hormone-insensitivity therapies in EC (116, 173). Metformin has been a well-tolerated biguanide drug to treat type 2 diabetes mellitus for decades. In the context of hyperinsulinemia easily accompanying EC patients, some studies have demonstrated that metformin could suppress the proliferation of ECCs by changing GLUT1-related glucose metabolism and inhibiting the PI3K/AKT/mTOR signaling pathway (174). Moreover, metformin could facilitate the expression of PR, which greatly promotes the sensitivity of medroxyprogesterone acetate (MPA)-induced apoptosis progestin in resistant ECCs (175). However, direct data concerning metformin plus progestin producing a better therapeutic effect than progestin alone have not been found. Its side effects include diarrhea, dyspepsia, and flatulence.

In addition, it has been reported that some flavonoids, such as flavones and phloretin, show a well-established inhibition of GLUT1 *via* against ERs (176, 177). This suggests that GLUT1 inhibitors seem to be more effective in ER-positive EC.

### Glucose transporter-related clinical trials

Until now, most advances in the GLUT inhibitors are in the early preclinical stage, while a few are in the clinical trial stages of many cancers except EC (148). Several indirect data still exist; yet in a phase I trial of glioblastoma, 2-DG performed notable effectiveness in asymptomatic QTc prolongation and restriction of dose escalation (178). In addition, 2-DG combined with radiation therapy shows improvement including better tolerance of 2-DG toxicity and lower incidences of late radiation effects in glioblastoma patients (179). There are as few as 20 ongoing clinical trials on curcumin combination therapy, with two being related to EC. Both are in phase II trials: one was completed in 2016, which showed an increased therapeutic effect on standard treatment through disturbing tumor-induced inflammation. However, the other trial concerning pembrolizumab, radiation, and immune modulation is still ongoing (180). Furthermore, in obese women with early EC, a phase II non-controlled trial has reported that metformin combined with MPA could lead to a better complete response (CR) rate and a recurrence rate than MPA alone (181). Another randomized controlled clinical trial also confirms that the addition of metformin is associated with a higher early CR compared with megestrol acetate (MA) alone (182). In a phase III CONFIRM clinical trial, the VEGFR inhibitor PTK787/ZK 222584 (vatalanib) has been confirmed to have greatly increased benefit as compared to original agents in metastatic colorectal cancer patients (183).

If combined with other radio- or chemo-therapeutic agents and hormone therapy, these inhibitors could become an excellent helper to enhance therapeutic sensitivity and reduce toxicity and dosage. In fact, there are many studies focusing on the combination of GLUT inhibitors with various glycolytic inhibitors (e.g., hexokinase 2 (HK2) inhibitors, PKM2 activators, and lactate dehydrogenase (LDH) inhibitors). Their joint functions to confront glycolytic and mitochondrial metabolism also make promising effectiveness in the treatment of active proliferative cancer. Thus, preclinical and clinical trials are needed for GLUT-related inhibitors and GLUT inhibitors to be used for EC patients.

## Perspectives

The main GLUTs in EC are Class 1 (GLUTs 1–4) and Class 3 (GLUTs 6, 8), and the overexpression of these GLUTs has been observed. As mentioned above, such abnormal overexpression of GLUTs may be related to the regulation of estrogen or progesterone, insulin and high glucose, microenvironment (such as hypoxia and cytokines), and so on. On the one hand, these overexpressed GLUTs provide abundant glucose uptake for various metabolic pathways; on the other hand, they also participate in the activation of many crucial signaling pathways and the regulation of key genes concerning proliferation and apoptosis, EMT, and angiogenesis in EC. In addition, overexpressed GLUTs may also cause ECCs to be insensitive to hormone therapy or even resistant to chemoradiotherapy, which has become a huge challenge in the treatment of EC in recent years. Nevertheless, from what has been discussed in this review, we can conclude that with more and more attention to the regulation of various GLUTs and GLUT-related inhibitors in EC, patients are bound to receive more effective treatment strategies and better outcomes. For future EC therapies, there is a consensus to monitor GLUT expression in tumors that are being treated with several appropriate therapies (e.g., hormonal, chemotherapy, and radiotherapy), to ascertain how their expression levels and activity change under these treatments.

However, there are still many problems that remain to be solved. For example, the specific mechanism of GLUTs in the regulation of endometrium, especially the heterogeneity of GLUTs in EC, has not been clarified yet. Breakthroughs in these fields will promote the development of personalized and precise treatment of EC. In addition, the regulation of GLUTs on the immune microenvironment in EC also deserves to be further studied. The expression characteristics and metabolic regulation mechanisms of GLUTs (e.g., GLUT1, GLUT4, GLUT6, and GLUT8) in the EC microenvironment are likely to be a hotspot, which will provide a basis for the realization of immunometabolism typing of EC. Last but not least, how GLUT inhibitors can reach maximum utilization in EC precision-targeted therapies also remains to be explored. In particular, how to achieve an efficient synergistic effect of GLUT inhibitors and hormone therapy may be a focus of future research.

## Author contributions

XZ and JJ-L drafted and revised the manuscript. AA, DY-H, JD, JN-W, and LB-L edited the manuscript. FX and MQ-L conceived and designed the review and edited the manuscript. All the authors were involved in writing the manuscript.

## Funding

This work was supported by the Major Research Program of the National Natural Science Foundation of China (NSFC, 82072872, 92057119, and 31970798), the Program for Zhuoxue of Fudan University (JIF157602), and the Support Project for Original Personalized Research of Fudan University (IDF157014/002).

## Conflict of interest

The authors declare that the research was conducted in the absence of any commercial or financial relationships that could be construed as a potential conflict of interest.

## Publisher’s note

All claims expressed in this article are solely those of the authors and do not necessarily represent those of their affiliated organizations, or those of the publisher, the editors and the reviewers. Any product that may be evaluated in this article, or claim that may be made by its manufacturer, is not guaranteed or endorsed by the publisher.
